# An investigation into religious awareness as a crucial factor in adherence to COVID-19 medical directives in Palestine

**DOI:** 10.1186/s12889-022-13767-9

**Published:** 2022-07-13

**Authors:** Munther Saeedi, Nihad Al-Othman, Maha Rabayaa, Saeed Dwaikat

**Affiliations:** 1 Language Centre, Faculty of Human Sciences, An-Najah National University, Nablus, State of Palestine; 2 Biomedical Department, Faculty of Medicine and Health Sciences, An-Najah National University, Nablus, State of Palestine; 3 Department of Osoul Aldeen, Faculty of Sharia, An-Najah National University, Nablus, State of Palestine

**Keywords:** COVID-19, Religious, Awareness

## Abstract

**Background:**

Coping with the pandemic caused by the SARS-COV- 2 has been a global challenge. To this end, several measures have been adopted to control the transmission of the disease and to ensure public safety. One factor that has greatly affected the community’s behaviors, attitudes, and practices in Palestine has been religious beliefs.

**Aim:**

This study aims to investigate the role of religion as a factor in adherence to the COVID-19 medical directives in Palestine.

**Methods:**

A descriptive cross-sectional study was performed from August to October 2021. In this study, 1,353 participants were asked to complete a questionnaire that consisted of 20 items that measured the impact of religious beliefs and the role played by religious scholars in the promotion and application of medically-approved health directives and the rectification of COVID-19 related information. The data were analyzed by using SPSS version 22 software.

**Results:**

More than 50% of the participants agreed that religion has a positive impact on community adherence to the health instructions in the majority of studied items. The responses were significantly variable based on the age and place of residence in most of the questions (*p*-value < 0.05). However, gender and to a lesser extent, the level of education affected the responses to many research aspects less significantly.

**Conclusions:**

Religion could be an effective tool in dealing with challenging health issues such as COVID-19. Intervention programs can be developed based on the community’s religious beliefs, attitudes, and practices, to dispel myths regarding the disease and to encourage community commitment and adherence to health directives.

## Background

In 2019, several cases of pneumonia of an unknown etiology were detected for the first time in China, which were then identified to be caused by the novel severe acute respiratory syndrome coronavirus -2 (SARS-CoV-2). Henceforth, the disease was named COVID-19 [1]. Since the identification of the initial cases, the number of SARS-CoV-2 cases has increased drastically worldwide, and on March 11, 2020, the World Health Organization (WHO) announced a global pandemic state [2]. Nowadays, the SARS-CoV-2 is affecting more than 200 countries, and the number of cases increased globally from less than a hundred thousand in February 2020 to more than 250 million cases, with more than 5 million deaths recorded by 7 November 2021, according to the American Library Association certified Worldometer. No one can deny the fact that the COVID-19 pandemic is one of the most serious pandemics that has challenged the human race at this time. It has resulted in many dire consequences not only for individuals or families but also for entire communities and countries. Nevertheless, it has gained an unprecedented interest and care in almost all sectors mainly pubic health sectors and religious ones. A lot of research has been conducted on COVID-19 from a purely medical perspective; however, little research has been done on the interplay between it and religion.

The interplay between religion and the pandemic started to draw attention at the beginning of 2020 first at Iran [3], South Korea [4], Southeastern Asian countries such as Malaysia [5], and after that in Africa [6]. Many gatherings and assemblies (e.g., congregations, funerals, marriages, births, baptisms, etc.) were held in religious sites and resulted in putting large numbers of people very close to each other making them “as a potential focal point for dispersal of novel pathogens” ([7] p. 219) mainly those which are airborne. Disease outbreaks and global spread are directly affected by such gatherings.

Religion has often been portrayed as a problem during the COVID-19 pandemic. The impact of religion on public health is either directly detrimental or indirectly deleterious. Therefore, exercising preventive measures becomes imperative to cut down on the likelihood of cross infections [7] and reduce outbreaks rates. Religion stimulates health and overall well-being; it bans a lot of detrimental acts such as adultery, committing suicide, taking drugs, drinking alcohol, etc. Besides, it helps a shift in focus from “pathogenetic” into “salutogenic” health-promoting paradigm [8]. It is particularly during stressful or anxious times that religion can serve as a coping mechanism. During the COVID-19 pandemic, many rituals have been modified and changed to promote social distancing and decrease gatherings to the minimum.

Religious awareness has been proved to be imperative in promoting health and maintaining well-being [7]. Religion substantially touches upon health from four different angles including the various health practices or behaviors, socialization and relationships, psychological well-being, and the functionality of the mind [8]. Two theoretical frameworks can be important for considering religion within public health generally and in the context of COVID-19. On the one hand, education scientists have argued that controversial issues such as evolution and vaccine hesitancy can be more effectively communicated to individuals who are likely to reject them if they are reframed and viewed as worldviews, rather than as manifest misconceptions, and if they are treated sensitively.

On the other hand, systems are considered complex with vague boundaries according to complexity theory. Complexity is “a dynamic and constantly emerging set of processes and objects that not only interact with each other but come to be defined by those interactions” [9]. Objects and processes, within the scope of this theory, are fundamentally interrelated, and as such reflexivity is important. As a result, it is easier to incorporate religion and examine how it may influence health in various ways. By incorporating religion into existing frameworks that draw on complexity theory, health promotion sensitively addresses religious aspects.

The unprecedented global health concern caused by COVID-19 has shown cooperative work between epidemiologists, scientists, politicians, educators, and healthcare workers to tackle the pandemic properly [10]. Since the beginning of the pandemic, several strategies have been adopted to prevent disease transmission; social distancing and lockdowns were prioritized in the times when the vaccine was not developed yet or made available in some countries [11]. However, the application of these strategies coincides with two major issues. First, they are unsustainable in the long term with the social, economical, religious, and psychological needs of the community [11]. Secondly, they were rejected by those in the population who were deeply suspicious of the protective measures and treatments advocated by the medical community and who had developed or had been influenced by alternative non-scientific theories regarding the disease [12, 13]. It should be taken into account that the previous experiences in other outbreaks such as Ebola have proven that health-promoting policies and disease preventive measures are worthless without taking into consideration the religious and cultural variabilities among communities since The lack of mutual trust between the health officials and the affected community would eventually worsen the outcomes [14, 15]. In the recent pandemic caused by the highly contagious SARS-CoV-2, raising awareness is a critical aspect of curbing the transmission of the disease effectively.

The role of religion in the social determinants of health is underestimated even though religion matters in public health [16]. Religion affects daily social activities globally [4]. Therefore, medical protocols which include banning large gatherings in mosques or churches for worship as a means of prevention of the transmission of COVID-19, are in opposition with the popular religious sentiment. This makes the role of religious scholars especially vital in instilling confidence in their congregations in the medical guidelines they are asked to observe [16, 17].

This study aims to investigate the role of religious awareness in making people stick to COVID19 medical directives in Palestine. The study investigated the impact of religious beliefs, the popular response to mandatory constraints upon religious activities, the role played by the government, religious leaders as well as social media in spreading awareness of the need to implement safety measures.

## Materials and methods

### Ethical consideration

This study received official ethical approval from the Institutional Review Board at An-Najah National University located in Nablus/Palestine. The study abided by “the Declaration of Helsinki (DOH).” All ethical considerations for medical research concerning human subjects were enforced. The confidentiality and the rights of the subjects of the study were preserved throughout the study. Written informed consent was provided and handed to each participant. The form described the study procedure, duration, benefit, and lack of any harmful intentions.

Moreover, the form indicated that all data collected would be used for research purposes only, while any information related to the participants would be kept confidential from all parties except the research investigators. The participants were fully informed that participation in the study was voluntary and that no penalty would be enforced in case of nonparticipation.

### Study sample

Following an explanation of the purpose of this cross-sectional study, Palestinian citizens were randomly selected and recruited in August and October 2021 to participate in the study. Upon signing the informed consent, the researchers adopted Jekel’s equation to estimate the nonprobability sample size. A minimum sample size of 384 was estimated based on the 0.5 probability of individuals following the religious instructions with a 95% confidence level. To eliminate the standard error of the mean and calculate the non-response rate, the researchers decided to increase the sample size. Ultimately, 1353 participants completed the questionnaire and participated in the study.

### Inclusion and exclusion criteria

The inclusion criteria included participants who have religious beliefs and the mental ability to participate in the study and those who have completed the questionnaire. The participants were from different age groups, residential areas (city, camp, or village), and levels of education as shown in Table 1. The exclusion criteria included individuals who refused to participate in the study and individuals with psychological and mental disabilities. The incomplete questionnaires were also excluded from the study.

**Table 1:** Demographic characteristics of the study sample (*n* = 1353)

Variable		Frequency	Percentage (%)
Gender	Male	627	46.3
	Female	726	53.7
Age group (years)	18–20	142	10.5
	21—40	781	57.7
	41 – 60	356	26.3
	More than 60	74	5.5
Place of residence	City	666	49.2
	Refugee camp	106	7.8
	Village	581	42.9
Level of education	Tawjihi or less	91	6.7
	Diploma	92	6.8
	Bachelor	864	64
	Graduated studies	305	22.5
COVID-19 infection	Yes	458	33.9
	No	895	66.1
COVID-19 vaccination	Yes	851	62.9
	No	502	37.1

### Study instrument

A self-administered questionnaire in Arabic was used for data collection and was distributed to the study population. The questionnaire was made up of two sections: sociodemographic factors including age, level of education, gender, place of residence, health information regarding COVID-19 infection, and vaccination. The second section included 20 questions about the role of religious instructions as a factor in adherence to COVID-19 medical directives from the Ministry of Health in Palestine. This section included four parts: religious beliefs, application of the health instructions on the religious activities, the role of religious figures, and religious awareness through social media, government officials’ employment of religion**,** and religious sermons. Due to the absence of a validated instrument that measures the role of religion in health practices and to ensure the validity of the study instrument, the questionnaire was given to five experts in the field of public health. There was an agreement among them regarding the content of the questionnaire. The questionnaire reliability was tested using Cronbach’s alpha and it was 0.93.

### Pilot study

A pilot study was conducted and administered on 30 people of different age groups to ensure the effectiveness of the questionnaire in terms of language, structure, relevance, responses, and the time needed to complete it.

### Statistical analysis

Statistics were analyzed using SPSS version 22; descriptive statistics were employed to analyze the sociodemographic characteristics. Using univariate analysis, variables related to religious instruction and sociodemographic variables were compared. To find out whether the association between the sociodemographic variables and religious instructions is statistically significant at *p* < 0.05, Chi-Square Test was used.

## Results

### Demographic characteristics of the study sample

The data were analyzed and tested for normality and found to be normally distributed. With the use of a suitable available sample composed of 1506 participants, 1353 had completed the questionnaire and were included in the study. The response rate was 89.8%. Around 53.7% of them were females, and 57.7% were from the age group 21 to 40 years (all participants age was from 18 years old and above), 42.9% of the participants were holding bachelor's degrees and 49.2% of them were from cities. Of the 1353 participants, 33.9% had been infected with COVID-19, and 62.9% had received the COVID-19 vaccine. Demographic data are shown in Table 1.

### Religious attitudes

The religious attitudes were included in 20 items demonstrating the various ways in which religious awareness contributed to the adherence to the COVID-19 related health instructions. Three items cover the impact of religious beliefs; Religious awareness has contributed to the knowledge that not complying with Covid-19 health-related instructions is suicidal (item 1), religious awareness has clarified that not complying with COVID-19 health-related instructions represents disobedience to God (item 2), and religious awareness has clarified that ignoring COVID-19 health-related instructions brings harm to others around us (item 3). The next three item concerns the impact of the application of health instructions on religious activities; Religious awareness has contributed to encouraging the public to observe social distancing during the performance of prayers and worship, thereby reducing the incidence of corona cases (item 4), religious awareness has contributed to encouraging the public to accept the idea of suspending religious congregations in mosques and churches (item 5), and the suspension of religious rituals such as Haj, Omra, or Christmas celebrations has contributed to reducing covid -19 infections (item 6). The role of religious figures was also investigated in items from 7 to 11 which correspond to their role in the people’s adherence to covid-19 health-related instructions for the prevention of corona, the public adherence to covid-19 preventive measures, the rebuttal of the conspiracy theory that accompanied the Corona outbreak, encouraging people to emulate and adhere to health instructions, and to accept the idea of suspending religious rituals such as Hajj, Omra, or Christmas celebrations to prevent infection, respectively. The last nine items explore the role of the general religious awareness during the Friday sermons and church preachings (item 12), in social media (item 13), TV shows on space channels and local radio (item 14), government officials’ employment of religion (item 15), the role of religion in clarifying the truth and refuting misinformation about COVID-19 (item 16), raising the public’s awareness of the gravity of COVID-19 related situation (item 17), encouraging the public to take COVID-19 vaccines (item 18), motivating health workers to make a greater effort to confront the Corona pandemic (item 19), and the acceptance of the idea of observing social distancing in weddings, during condolence visits, and other social events (item 20), shown in Table 2.

**Table 2:** The proportion of different religious attitudes toward COVID-19 among the study sample in Palestine (*n* = 1353)

Question	Strongly agree n (%)	Agree n (%)	I do not know n (%)	Disagree n (%)	Strongly disagree n (%)	Gender (*p-*value)	Age groups (*p-*value)	Level of education (*p-*value)	Place of residence (*p-*value)
**Religious beliefs**									
1. Religious awareness has contributed to the knowledge that not complying with Covid-19 health-related instructions is suicidal	193 (14.3%)	606 (44.8%)	266 (19.7)	237 (17.5%)	51 (3.8%)	0.000	0.000	0.153	0.009
2. Religious awareness has clarified that not complying with COVID-19 health-related instructions represents disobedience to God	214 (15.8%)	631 (46.6%)	237 (17.5%)	211 (15.6%)	60 (4.4%)	0.000	0.000	0.004	0.000
3. Religious awareness has clarified that ignoring COVID-19 health-related instructions brings harm to others around us	561 (41.5%)	621 (45.9%)	71 (5.2%)	71 (5.2%)	30 (2.2%)	0.246	0.000	0.008	0.000
**Application of the health instructions on the religious activities**									
4. Religious awareness has contributed to encouraging the public to observe social distancing during the performance of prayers and worship, thereby reducing the incidence of corona cases	248 (18.3%)	753 (55.7%)	145 (10.7%)	154 (11.4%)	53 (3.9%)	0.046	0.000	0.445	0.000
5. Religious awareness has contributed to encouraging the public to accept the idea of suspending religious congregations in mosques and churches	146 (10.8%)	625 (46.2%)	195 (14.4%)	290 (21.4%)	97 (7.2%)	0.103	0.001	0.211	0.000
6. The suspension of religious rituals such as Haj, Omra, or Christmas celebrations has contributed to reducing covid -19 infections	160 (11.8%)	518 (38.3%)	198 (14.6%)	311 (23.0%)	166 (12.3%)	0.240	0.021	0.214	0.033
**The role of religious figures**									
7. Appeals and statements by religious figures have contributed to people’s adherence to covid-19 health-related instructions for the prevention of corona	163 (12.0%)	699 (51.7%)	243 (18.0%)	205 (15.2%)	43 (3.2%)	0.041	0.000	0.269	0.000
8. The guidance of Religious scholars has made the public adhere more to covid-19 preventive measures	148 (10.9%)	670 (49.5%)	300 (22.2%)	193 (14.3%)	42 (3.1%)	0.000	0.000	0.656	0.000
9. The numerous Fatwas and appeals of religious scholars have contributed to the rebuttal of the conspiracy theory that accompanied the Corona outbreak	102 (7.5%)	501 (37.0%)	414 (30.6%)	256 (18.9%)	80 (5.9%)	0.000	0.000	0.018	0.000
10. The public appearance of religious figures adhering to prevention measures has encouraged people to emulate and adhere to health instructions	142 (10.5%)	591 (43.7%)	330 (24.4%)	240 (17.7%)	50 (3.7%)	0.006	0.000	0.009	0.000
11. Under the guides of religious scholars, the public has been encouraged to accept the idea of suspending religious rituals such as Hajj, Omra, or Christmas celebrations to prevent infection	163 (12.0%)	605 (44.7%)	189 (14.0%)	296 (21.9%)	100 (7.4%)	0.172	0.000	0.060	0.036
**Religious awareness through social media, government officials’ employment of religion, and during religious activities**									
12. Friday sermons and church preachings have contributed to encouraging people to adhere to covid-19 health-related instructions, and so helped reduce the incidence of injuries and deaths	181 (13.4%)	627 (46.3%)	335 (24.8%)	171 (12.6%)	39 (2.9%)	0.000	0.006	0.074	0.000
13. Religion-related posts on social media have contributed to greater adherence to covid-19 health-related instructions to prevent the disease	164 (12.1%)	709 (52.4%)	243 (18.0%)	199 (14.7%)	38 (2.8%)	0.125	0.000	0.505	0.000
14. Religion-related TV shows on space channels and local radio have contributed to raising public awareness of COVID-19 health-related instructions and so reduced the incidence of the corona	145 (10.7%)	672 (49.7%)	288 (21.3%)	204 (15.1%)	44 (3.3%)	0.010	0.000	0.446	0.000
15. The use of religious terms and phrases by the government officials has contributed positively to influencing people to adhere to health instructions to counter the Corona pandemic	156 (11.5%)	673 (49.7%)	267 (19.7%)	202 (14.9%)	55 (4.1%)	0.002	0.084	0.999	0.000
16. Religious awareness has contributed to clarifying the truth and refuting misinformation about COVID-19	92 (6.8%)	610 (45.1%)	360 (26.6%)	229 (16.9%)	62 (4.6%)	0.018	0.000	0.004	0.000
17. Religious awareness has contributed to raise the public's awareness of the gravity of COVID-19 related situation	210 (15.5%)	639 (47.2%)	162 (12.0%)	249 (18.4%)	93 (6.9%)	0.953	0.001	0.147	0.010
18. Religious awareness has contributed to encouraging the public to take COVID-19 vaccines as a form of prevention	119 (8.8%)	607 (44.9%)	332 (24.5%)	242 (17.9%)	53 (3.9%)	0.017	0.000	0.207	0.000
19. Religious rhetoric has had a major impact in motivating health workers to make a greater effort to confront the Corona pandemic	166 (12.3%)	592 (43.8%)	358 (26.5%)	178 (13.2%)	59 (4.4%)	0.361	0.000	0.263	0.000
20. Religious awareness has contributed to people’s acceptance of the idea of observing social distancing in weddings, during condolence visits, and other social events	138 (10.2%)	678 (50.1%)	187 (13.8%)	283 (20.9%)	67 (5.0%)	0.370	0.000	0.123	0.000

The five-point Likert scale was used to reveal the proportion of answers on the different religious attitudes during the COVID-19 pandemic. The majority of the study sample agree that religious awareness has influenced the community’s adherence to the COVID-19 medical directives during the pandemic. More than 50% of the study sample either agree or strongly agree on all items except item 9 (44.5%). Less than 30% of the study sample responses either disagreed or strongly disagreed on all items. The most influencing items were item 3 in which more than 85% were in agreement with the role of religious awareness in clarifying that ignoring COVID-19 health-related instructions brings harm to others around us and then item 4 in which more than 70% were also in agreement with the role of awareness during religious activities. Shown in Table 2.

## The impact of demographic factors on the religious attitudes

### Religious beliefs

Items 1, 2, and 3 connect the nonadherence to the COVID-19 health-related instructions with the basic religious beliefs, such as the commission of suicide, disobedience to God, and harming others, respectively. There was a significant difference in responses to the first item between genders, different age groups, and the place of residence (*p*-value < 0.05). However, the levels of education did not significantly affect the responses (*p*-value > 0.05). The responses to item 2 were significantly variable between genders, age groups, level of education, and place of residence (*p*-value < 0.05). For item 3, there were significant differences in responses among different age groups, levels of education, and places of residence (*p*-value < 0.05) while it was not significant between genders.

### Application of the health instructions on the religious activities

Items 4, 5, and 6 revealed the role of religious awareness in accepting social distancing during religious activities, banning religious congregations in mosques and churches, and suspending religious rituals to reduce the incidence of the disease, respectively. The responses to item 4 were significantly variable in terms of gender, age, and place of residence (*p*-value < 0.05). The responses to items 5 and 6 were significantly variable in terms of age and place of residence (*p*-vale < 0.05). It was observed that the level of education and gender did not significantly affect the responses regarding the application of health-related instruction in religious activities.

### The role of religious figures

The items from 7 to 11 represent the role of religious leaders: Item 7 (appeals and statements by religious figures have contributed to people’s adherence to health-related instructions for the prevention of covid-19); item 8 (religious scholar’s guidance has influenced the public to adhere more to Covid-19 preventive measures); item 9 (the numerous Fatwas and appeals of religious scholars have contributed to the rebuttal of the conspiracy theory that followed the Corona outbreak); item 10 (religious figures who publicly adhered to the preventive measures as a means to encourage people to emulate the example); and item11 (The contribution of religious scholars in encouraging the public to accept the suspension of religious rituals such as Hajj, Omra, or Christmas celebrations to prevent the spread of the infection), these items shed light on the extent of the influence of religious leaders through their sermons and activities in educating the public about COVID-19, the safety guidelines, and encouraging its cooperation in adhering to them. The responses to items from 7 to 11 were significantly variable in terms of age and place of residence (*p*-value < 0.05). Based on gender, the responses were significantly variable regarding the role of religious figures except for item number 11. The level of education was the least variable that significantly affected the responses. It was only significant in items 9 and 10 (*p*-value < 0.05).

## Religious awareness through social media, government officials’ employment of religion, and religious activities

Items from 12 to 20 covered the various aspects of religious awareness that were carried out in religious places (item 12), on social media (items 13 and 14), and during the government official's employment of religion (item 15) to promote adherence of the community to the health directives. Moreover, religious awareness had been used to refute the misinformation about COVID-19 (item 16), raise the public awareness of the gravity of the COVID-19 (item 17), encourage people to take the COVID-19 vaccine (item 18), motivate the healthcare workers (item 19), and to increase the community acceptance of distancing during social activities (item 20). In terms of gender, the significant difference in responses regarding these items (from 12 to 20) was observed in items 12, 14, 15, 16, and 18. Except for item 15, the responses to items from 12 to 20 significantly varied between different age groups. There was no significant difference in responses based on the level of education except for item 16 concerned the refutation of misinformation regarding COVID-19. The place of residence significantly affected the responses to all the items from 12 to 20 (*p*-value < 0.05).

## Discussion

This study investigated the incorporation of religion as a means to influence people to follow COVID-19 protocols in Palestine. Since the beginning of the COVID-19 pandemic in 2020, the disease has transmitted rapidly all over the world, affecting all aspects of life, including health, economy, psychology, and social life. Several evidence-based guidelines have been applied by governments globally to decrease the viral spread and to improve the community's wellbeing. These strategies, such as lockdowns, distancing, isolations, testing, and vaccinations, have been announced and applied. However, community acceptance and adherence to these health-related instructions were a critical challenge.

Addressing the patients’ religious beliefs during medical care is important and had been proven in dealing with other infectious diseases and suicidal behaviors [18]. While other studies have shown a decreased adherence to the COVID-19 mitigation policies in communities with higher religiosity [19]. In our study, a high level of consensus has been observed regarding the role of religious awareness in accepting the social distancing during religious activities, suspending religious congregations in mosques and churches, and participating in religious rituals such as Haj, Omra, or Christmas celebrations, as a means to reduce disease transmission. A high level of compliance is reported in terms of social distancing and suspension of religious activities that gather a large number of the population simultaneously [20]. These practices are essential during the current situation because the gathering of different nationalities during religious rituals, such as Haj, is dangerous and will worsen the pandemic because SARS-CoV-2 has shown rapid evolution and highly transmissible mutations that can span rapidly around the world.

The widespread reliance on conspiracy theories regarding COVID-19 has affected adherence to health-related instructions and the acceptance of diagnosis and treatment [21]. Our study shows an agreement about the role of religious figures in rebutting these conspiracy theories regarding the COVID-19, which have also been elicited in other countries [22]. This may be explained by the role of religiosity in mediating trust in science which represents some part in community acceptance and adoption of health-related strategies [23].

A previous study revealed a significant positive correlation between the awareness, attitudes, and practices concerning COVID-19 [24]. This proves the importance of organizing proper awareness programs that target the community based on gender, age, educational level, and place of residence, and this is supported in our study since significant variations in responses based on these demographic variables have been revealed [25, 26]. As people get older, they are more likely more religious especially if they realize that that could have some positive influence on their health. As result, their response to awareness that is related to religion becomes more noticeable. Also, it is worth mentioning that old people tend to be more vulnerable to covid 19 according to WHO. As for the place of residence, cities are often a place of attraction to people coming from different residential areas such as villages and camps, so city residents are more likely to be responsive to medical directives as the possibility of infection is high. Besides, cities are home to a great number of worshipping places such as mosques and churches. As a result, the role of religious awareness is more likely to be more significant.

It has been reported that there is a gap in the knowledge, attitudes, and practices concerning the COVID-19 pandemic, such as social distancing and the wearing of protective masks [26]. Our study reported a high agreement from the community regarding the vital role of various aspects of religious awareness through social media, governmental officials, and participation in less risky religious activities in line with the health instructions. Other studies have shown the importance of the harmony between religious, community, and educational leaders in promoting awareness, increasing community compliance with health protocols, overcoming the vaccination hesitancy, and provoking trust in science [22, 27, 28].

The observed role of religion in helping the public cope with the COVID-19 pandemic proves what has been previously reported about dealing with health crises. A multidisciplinary approach, where culture, religion, and the state collaborate, should be adopted to mitigate the virus transmission and maintain public health during the pandemic [29–31]. Furthermore, an inverse correlation between the mitigating effect of religion and adults’ mental health problems was revealed in another study [32] and this proves the importance of integrating religion as a major discipline in dealing with health crises such as COVID-19. Furthermore, statistically we found that vaccinated people had a more positive attitude to medical directives thanks to the impact of religious awareness. (Data are not shown).

## Conclusions and recommendations

This study has revealed that popular religious beliefs regarding obedience to God, non-compliance with COVID-19 health guidelines being tantamount to the commission of suicide, maintaining the safety of others are influential in the public’s acceptance and adherence to the safety measures. Additionally, the impact of religious awareness on these areas varied according to gender, age group, level of education, and place of residence. Furthermore, Our results indicate that religious figures play a vital role in promoting adherence to health-related instructions either by statements, appeals, or personal examples. Generally, owing to religious awareness in its different forms, it was found that people adhered to COVID-19 health directives. Worshippers wore their masks and observed social distancing especially when they were performing religion-related ceremonies. In addition, we found that religious figures have a great effect on health behaviors.

The implementation of health directives should take into account religion as a vital aspect of the community’s daily activities, attitudes, and beliefs. Introducing health directives by religious scholars through religious activities could improve the community’s commitment to health-related instructions. Proper health awareness campaigns that target different genders, age groups, residential areas, and levels of education should be further studied and developed.

## Data Availability

All the utilized data to support the findings of the current study are included in the article.

## Abbreviations

COVID-19: Coronavirus disease of 2019
DOH: Declaration of Helsinki
CDC: Center of Disease Control and Prevention
SARS-CoV-2: Severe acute respiratory syndrome coronavirus-2
WHO: World Health Organization
